# Height loss but not body composition is related to low back pain in community-dwelling elderlies: Shimane CoHRE study

**DOI:** 10.1186/s12891-019-2580-6

**Published:** 2019-05-10

**Authors:** Takeshi Endo, Takafumi Abe, Kenju Akai, Tsunetaka Kijima, Miwako Takeda, Masayuki Yamasaki, Minoru Isomura, Toru Nabika, Shozo Yano

**Affiliations:** 1 Division of Internal Medicine, Unnan City Hospital, Unnan-city, Shimane Japan; 2 Center for Community-Based Healthcare Research and Education (CoHRE), Shimane University, Matsue-city, Shimane Japan; 3 Department of General Medicine, Shimane University Faculty of Medicine, Izumo-city, Shimane Japan; 4 Shimane University Faculty of Human Sciences, Matsue-city, Shimane Japan; 5 Department of Functional Pathology, Shimane University Faculty of Medicine, Izumo-city, Shimane Japan; 6 Department of Laboratory Medicine, Shimane University Faculty of Medicine, Izumo-city, Shimane Japan

**Keywords:** Lumbago, Health examination, Geriatrics, Muscle mass

## Abstract

**Background:**

Low back pain (LBP) is a common complaint in the elderly Japanese population. Although previous studies showed that height loss was associated with LBP, it remains unclear whether LBP is associated with body composition. The objective of the present study was to investigate whether body composition and physical characteristics, including height loss, were associated with LBP.

**Methods:**

The present study is retrospectively registered, and the participants were 2212 community-dwelling Japanese people aged over 60 years who participated in the Shimane CoHRE study in 2016. We investigated the presence of LBP, body composition parameters (muscle, fat, body weight, and bone mass), physical characteristics (body height and height loss), chronic diseases, history of fall, smoking, and drinking habits. We examined the relationships of body composition parameters and physical characteristics with point prevalence of LBP using multivariate logistic regression.

**Results:**

The point prevalence of LBP was 43.2% in women and 39.5% in men. Logistic regression models showed that body height and body composition were not significantly associated with LBP; however, height loss was associated significantly with LBP in women and men (OR: 1.14, 95% CI: 1.08–1.20 and OR: 1.13, 95% CI: 1.06–1.21, respectively). Hypertension (OR: 1.32, 9 5% CI: 1.04–1.69) and chronic heart disease (OR: 1.57, 95% CI: 1.01–2.43) in women and history of fall (OR: 1.70, 95% CI: 1.13–2.56) and cerebrovascular disease (OR: 1.88, 95% CI: 1.05–3.34) in men were significantly associated with LBP. However, body composition was not associated with LBP in either gender.

**Conclusions:**

The present study demonstrated that height loss, but not body composition, was related to LBP in community-dwelling elderly people. To elucidate the cause of LBP, it is important to consider the relationship with height loss.

**Electronic supplementary material:**

The online version of this article (10.1186/s12891-019-2580-6) contains supplementary material, which is available to authorized users.

## Background

Low back pain (LBP), a common symptom of the elderly, is associated with a marked decrease in health-related quality of life [1, 2] and physical function later in life [3–5]. Furthermore, LBP cause economic burdens on individuals, communities and governments, including direct costs for health-care and indirect costs of lost production and lost household productivity [6–9].

A previous survey reported that the point prevalence of LBP ranged from 12 to 33%, 1-year prevalence ranged from 22 to 65%, and lifetime prevalence ranged from 11 to 84% [10]. In the Japanese population, the point prevalence of LBP was reported to be 37.7% [11], suggesting that Japanese people have considerably higher rates of LBP than do individuals in other parts of the world. Therefore, prevention of LBP is an important issue for Japanese public health.

Many researchers have studied the physical characteristics of subjects with LBP. For example, LBP has been reported to be associated with overweight/obesity [12–18] and substantial body height [19–22]. On the other hand, height loss has been reported to be associated with LBP [23, 24]. Furthermore, some have reported a relationship of LBP with high fat mass [25–27], while others have shown a relationship between LBP and muscle atrophy [28–30]. However, to the best of our knowledge, there have been no reports examining the relationship of LBP with body composition and height loss.

Therefore, the aims and objectives of the present study were to examine the relationship between body composition, physical characteristics, and LBP with respect to height loss in general community-dwelling older people.

## Methods

### Study design

This cross-sectional study is a part of the cohort study conducted by the Center for Community-based Healthcare Research and Education in Shimane University (Shimane CoHRE study). It is an ongoing health examination for the community-dwelling people in Unnan-city, Okinoshima-cho and Ohnan-cho, Shimane Prefecture, Japan. The study protocol was approved by the Ethics Committee of Shimane University School of Medicine (#3149) and Unnan City Hospital (#20180004). Written informed consent was obtained from all participants.

### Study participants

**Fig. 1:**
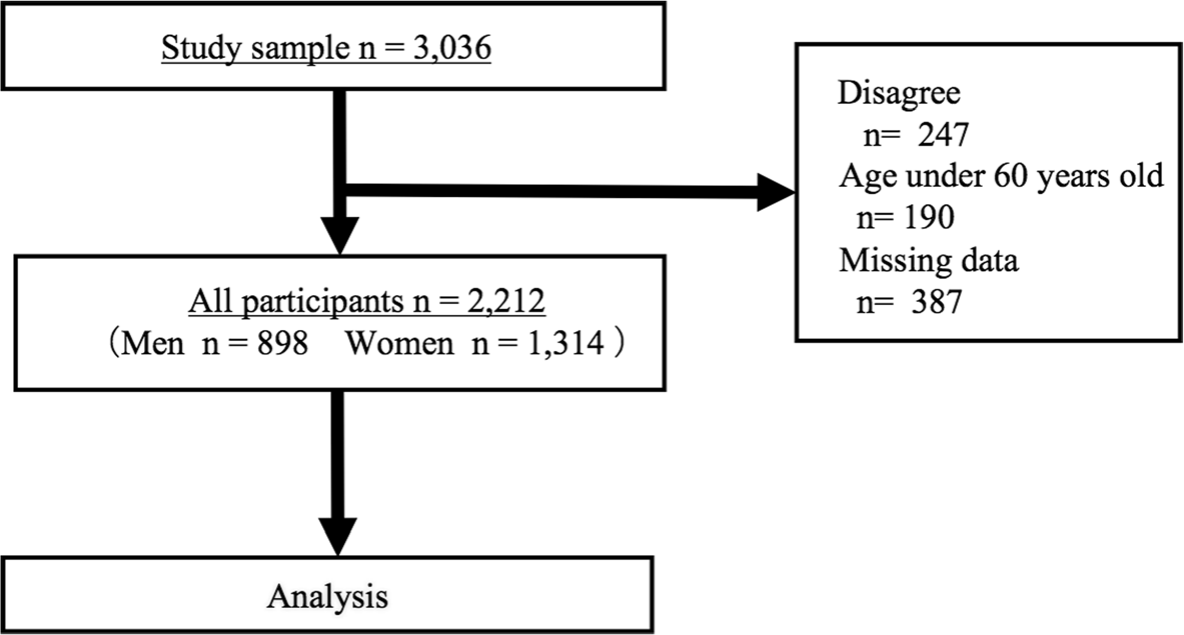
Study flow

In this study, we used the CoHRE study data, which was collected from June to November 2016. A total of 3036 community-dwelling Japanese people participated in the health examinations. The inclusion criteria for this study were as follows: (1) individuals who were over 60 years old; (2) individuals answered the questionnaire regarding LBP and history of fall; (3) individuals whose demographic data, including age, gender, and the tallest recalled height were recorded; (4) individuals whose body compositions were examined; and (5) individuals who were informed of the protocol and purpose of the current study and consented to participate. According to the criteria, a total of 2212 subjects were included in the current study (Fig. 1).

### Low back pain

The presence of LBP was assessed using self-reported questionnaire and face-to-face information. Participants were asked the following question: ‘Do you have any low back pain at present: yes or no?’ Those who answered ‘yes’ were defined as having LBP. This method was used in a previous study [31].

### Body composition parameters

#### Muscle, fat, body weight and bone mass

Body composition and body weight were measured by bioelectrical impedance analysis (BIA) method with MC-780A multi-frequency segmental body composition analyzer (Tanita Co., Tokyo, Japan) [32, 33]. Body fat mass (kg), body fat ratio (%), muscle mass of the arms and the legs (kg), and body mass index (BMI) were automatically calculated.

Bone mass was measured using quantitative ultrasound (QUS) (Benus α; Ishikawa Seisakusho, Ltd., Ishikawa, Japan). QUS has advantages including absence of exposure to radiation, low cost, and portability. The estimated values were compared to young adult mean (%YAM) of the same gender. A value 100% means same value as healthy young men or women [34]. This measurement was performed in all areas except Okinoshima-cho.

### Physical characteristics

#### Body height and height loss

The present body height was measured using a stadiometer. The tallest body height was obtained from self-reported questionnaire as follows: “What was your tallest height (when you were 20 years old)?” Height loss was calculated by the subtraction of the present height from the tallest height [24, 35].

### Covariates

Other variables were obtained from the self-administered questionnaires. We inquired about age, gender, history of fall within 1 year (yes, no), smoking (yes, no), alcohol consumption (yes, no) and chronic disease (hypertension, dyslipidemia, diabetes, cerebrovascular disease, chronic heart disease: yes, no). These potential confounders were reported to be associated with musculoskeletal pain, including LBP [36–42].

### Statistical analysis

The characteristics of study participants were compared between participants with LBP and those without using independent *t-*tests for continuous variables and χ^2^ tests for categorical variables. Although some data were not normally distributed, this analysis method was used because the number of participants was sufficiently large.

Multivariate logistic regression models stratified by gender were used to explore the association between the presence of LBP (dependent variable), body composition parameters, physical characteristics, and covariates (independent variables). According to these models, adjusted odds ratios (ORs) and 95% confidence intervals of the LBP-related parameters were calculated. ORs were adjusted for age, body composition, and additional covariates such as body characteristics and the presence of chronic disease. We used for the following potential confounders: age, body height, muscle mass, fat mass, smoking, alcohol drinking, history of fall, hypertension, dyslipidemia, diabetes, cerebrovascular disease, chronic heart disease, and height loss. Body weight and BMI were not included as dependent variables of multivariate analysis because of their high Pearson’s correlation coefficients with muscle mass and fat mass (Additional file 1: Table S1). We performed a multiple linear regression analysis to identify anthropometric parameters that were related to height loss, expressed as standardized β and 95% confidence interval (CI).

The level of significance was set at *p* < 0.05. All data were presented as the mean ± standard deviation (SD). All statistical analyses were performed using the IBM SPSS Statistics 22 software package (IBM Japan, Tokyo, Japan).

## Results

### Clinical characteristics of the study population

The characteristics of 2212 participants are shown in Table 1. The number of participants with LBP was 567 (43.2%) women and 355 (39.5%) men. There was no significant difference in the prevalence of LBP between women and men. Women with LBP were significantly younger and shorter and showed greater height loss and higher prevalence of hypertension and heart disease compared to those without LBP. Men with LBP showed greater height loss and higher rates of history of falls compared to those without LBP. Conversely, body composition and bone mass were not significantly different with or without LBP in men and women. Furthermore, no significant difference was found in the prevalence of LBP among the three communities in both genders.

**Table 1:** Baseline characteristics of study participants according to low back pain

	Women					Men				
	No LBP		LBP		*p*-value	No LBP		LBP		p-value
	*n* = 747		*n* = 567			*n* = 543		*n* = 355		
	Mean	SD	Mean	SD		Mean	SD	Mean	SD	
Age(years)	72.5	6.6	73.5	6.5	**0.01**	72.9	6.6	72.8	6.4	0.92
Body weight(kg)	50.8	8.4	50.5	8.3	0.52	60.5	8.6	61.0	8.9	0.40
Body height (cm)	150.6	5.9	149.1	5.7	**< 0.01**	163.1	6.1	162.7	6.2	0.42
Body mass index (kg/m^2^)	22.4	3.4	22.7	3.4	0.11	22.7	2.8	23.0	2.8	0.16
Muscle mass(kg)	14.2	2.0	14.0	2.1	0.21	20.2	2.9	20.4	3.2	0.25
Fat mass(kg)	15.2	6.4	15.4	6.2	0.55	11.7	5.0	11.9	5.1	0.51
Bone mass %YAM(%)^a^	83.9	10.0	83.4	9.9	0.40	91.3	11.0	91.3	12.3	0.93
Height loss (cm)	2.9	2.4	4.0	3.3	**< 0.01**	2.3	2.0	2.9	2.6	**< 0.01**
	n	%	n	%		n	%	n	%	
Alcohol intake	213	28.5	144	25.4	0.21	385	70.9	248	69.9	0.74
Current smoking	8	1.1	5	0.9	0.73	79	14.5	62	17.5	0.24
History of fall	113	15.1	96	16.9	0.38	56	10.3	58	16.3	**< 0.01**
Hypertension	279	37.3	259	45.7	**< 0.01**	212	39.0	156	43.9	0.14
Diabetes mellitus	68	9.1	45	7.9	0.46	77	14.2	49	13.8	0.87
Dyslipidemia	240	32.1	195	34.4	0.39	97	17.9	67	18.9	0.70
Cerebrovascular disease	11	1.5	15	2.6	0.13	24	4.4	28	7.9	**0.03**
Chronic Heart disease	42	5.6	55	9.7	**< 0.01**	62	11.6	34	10.7	0.38
Community 1	317	42.4	218	38.4		243	44.8	186	52.4	
Community 2	255	34.1	210	37.0		152	28.0	87	24.5	
Community 3	175	23.4	139	24.5	0.34	148	27.3	82	23.1	0.08

^a^*n* = 1505; Community 1 = Unnan city; 2 = Oki-island; 3 = Ohnan Cho

*S.D* standard deviation

### Association between low back pain and covariates

Next, we conducted multivariate logistic regression models for the presence of LBP in women and men. In women, body height (OR: 0.96, 95% CI: 0.93–0.98, *p* < 0.01) was negatively associated with the presence of LBP in Model 1 (Table 2). In model 2, however, height loss (OR: 1.14, 95% CI: 1.08–1.20, *p* < 0.01) but not body height was significantly associated with LBP. Furthermore, the presence of hypertension (OR: 1.32, 95% CI: 1.04–1.69, *p* = 0.02) and chronic heart disease (OR: 1.57, 95% CI: 1.01–2.43, *p* = 0.04) had a significant association with LBP in women. In men, height loss (OR: 1.13, 95% CI: 1.06–1.21, *p* < 0.01), history of fall (OR: 1.70, 95% CI: 1.13–2.56, *p* = 0.01) and cerebrovascular diseases (OR: 1.88, 95% CI: 1.05–3.34, *p* = 0.03) were significantly associated with LBP (Table 3).

**Table 2:** Odds ratios for low back pain in women (*n* = 1314) by multivariate logistic regression models

	Model 1					Model 2				
	OR	95% CI			p-value	OR	95% CI			p-value
Age	1.00	0.98	–	1.02	0.77	0.99	0.97	–	1.01	0.19
Body height	0.96	0.93	–	0.98	**< 0.01**	0.98	0.95	–	1.00	0.08
Muscle mass	1.02	0.95	–	1.11	0.55	1.01	0.93	–	1.10	0.74
Fat mass	1.00	0.98	–	1.02	0.85	1.00	0.98	–	1.02	0.95
Smoking	0.90	0.29	–	2.79	0.85	0.89	0.28	–	2.80	0.84
Alcohol	0.90	0.70	–	1.16	0.43	0.91	0.70	–	1.18	0.47
History of fall	1.05	0.77	–	1.42	0.77	1.05	0.77	–	1.43	0.76
Hypertension	1.31	1.03	–	1.66	**0.03**	1.32	1.04	–	1.69	**0.02**
Dislipidemia	1.05	0.82	–	1.35	0.67	1.11	0.87	–	1.43	0.39
Diabetes	0.73	0.48	–	1.09	0.13	0.73	0.48	–	1.11	0.14
Cerebrovascular disease	1.72	0.77	–	3.85	0.18	1.82	0.81	–	4.09	0.15
Chronic heart disease	1.61	1.05	–	2.48	**0.03**	1.57	1.01	–	2.43	0.04
Height loss	–	–		–	–	1.14	1.08		1.20	**< 0.01**

**Table 3:** Odds ratios for low back pain in men (*n* = 898) by multivariate logistic regression models

	Model 1					Model 2				
	OR	95% CI			p-value	OR	95% CI			p-value
Age	1.00	0.97	–	1.02	0.83	0.99	0.96	–	1.01	0.25
Body height	0.98	0.95	–	1.00	0.10	0.99	0.97	–	1.02	0.67
Muscle mass	1.05	0.99	–	1.12	0.11	1.04	0.98	–	1.11	0.20
Fat mass	1.00	0.96	–	1.03	0.76	1.00	0.96	–	1.03	0.82
Smoking	1.26	0.86	–	1.84	0.23	1.21	0.83	–	1.77	0.32
Alcohol	0.97	0.72	–	1.31	0.83	0.95	0.70	–	1.29	0.75
History of fall	1.74	1.16	–	2.61	**0.01**	1.70	1.13	–	2.56	**0.01**
Hypertension	1.19	0.89	–	1.61	0.24	1.21	0.90	–	1.63	0.21
Dislipidemia	1.00	0.70	–	1.44	1.00	1.07	0.74	–	1.54	0.72
Diabetes	0.89	0.59	–	1.33	0.56	0.92	0.61	–	1.38	0.69
Cerebrovascular disease	1.80	1.01	–	3.18	**0.04**	1.88	1.05	–	3.34	**0.03**
Chronic heart disease	0.75	0.47	–	1.19	0.22	0.75	0.47	–	1.19	0.22
Height loss	–	–		–	–	1.13	1.06		1.21	**< 0.01**

On the other hand, it was notable that no significant association was observed between LBP and body composition, including muscle mass and fat mass in both women and men. Furthermore, in a multiple linear regression analysis, height loss was associated inversely with height but was positively associated with weight in women (Additional file 2: Table S2).

## Discussion

The objective of the present study was to investigate whether body composition and physical characteristics were associated with the presence of LBP. We found that LBP was significantly associated with height loss, independent of body height, body composition, lifestyle, history of fall or chronic diseases. These findings suggest that height loss may be a good predictor of the presence of LBP in the elderly, at least in the Japanese population. Furthermore, height loss-related skeletal degeneration/disorders presumably account for many mechanisms of LBP.

In the present study, body height was negatively associated with the presence of LBP in women. However, this is inconsistent with findings of previous studies that suggested a positive association between body height and LBP [19–22]. Body height was an independently positive risk factor for herniated lumbar disc [43]. Therefore, we adjusted by height loss to show that height loss, but not present body height, was significantly and independently associated with LBP in both genders. With aging, height loss progresses [44], related to vertebral fractures, reduced disk hydration, change of vertebral deformities, and decrease in lumbar disc height [45–50]. Chronic pressure on the spine, such as from obesity or occupational physical loading, is thought to be one of the causes of height loss [46, 51]. Urquhart et al. demonstrated an association between obesity and reduced lumbar disc height [52]. Our study also showed a positive association between body weight and height loss in women, even if body height was considered.

In general, it is sometimes difficult to identify the specific pathology or causal disorder among subjects with LBP [53–55]. Nevertheless, the relationship between height loss and LBP was reported around two decades ago. Huang et al. reported a positive correlation between the number of vertebral fractures and the degree of back pain [56]. Ismail et al. reported an association between vertebral deformities and LBP and significant relationship between the number of deformed vertebrae and height loss [23]. It was thought that overloading the lumbar intervertebral discs may cause height loss and LBP [57]. Indeed, in a study of Japanese people, lumbar compression fractures and lumbar disc herniation were shown to be likely causes of LBP [58]. Another mechanism may be that shortened para spinal muscles may compress an intervertebral disk, leading to nerve root compression and pain [59]. Taken together, spinal degeneration or vertebral compression fracture, both highly related to height loss, appear to be major causes of LBP. In this regard, obesity has been thought to be linked to LBP [16–18, 20, 21, 25–27]. Livshits et al., in a study of twin women, reported that the risk factors of LBP were obesity and lumbar disc degeneration [60]. However, in the present study, body weight and BMI were not associated with LBP, suggesting that obesity might not be a major cause of LBP, at least in elderly Japanese people. For this reason, our subjects presumably showed much lower prevalence and/or milder degree of obesity, compared with those of other reports.

Regarding body composition, we did not find a significant association between LBP and muscle mass or of fat mass. These results were consistent with findings reported by Iizuka et al. [31]. Nevertheless, conflicting findings have been reported regarding an association between muscle mass and LBP. Some researchers showed a significant association of muscle mass reduction with LBP [24, 26, 27, 60], while others reported no association [25, 61, 62]. On the other hand, previous reports have shown positive associations between fat mass and LBP [24, 25]. Hussain et al. analyzed women with mean age of 50 years old, and showed a negative association of fat-free mass with LBP intensity [26]. Urquhart et al. reported a positive association between % fat and LBP in subjects with mean age of 47 years old [27, 61]. Because fat tissue secretes cytokines such as tumor necrosis factor and interleukin-6 [62], higher the body fat percentage and central adiposity correlates with higher levels of acute phase inflammatory proteins such as C-reactive protein [63, 64]. Furthermore, adipose mass and central adiposity were regarded as risk factors for knee and hip joint replacement [65, 66].

There are several limitations in this study. First, it is impossible to infer a causal relationship between LBP and the related factors, because this was a cross-sectional study. For example, the presence of LBP may lead to height loss due to difficulties of an extension of back muscle. On the other hand, LBP probably results from height loss because of skeletal disorders such as compression fractures, disc herniations, kyphosis, and degenerative spine disorders. Therefore, further studies are needed to analyze the causal relationship between LBP and physical characteristics, body composition, and chronic diseases. Second, we did not consider the severity, duration, and site of LBP. These factors may affect the relationship of LBP with height loss. Third, because tallest height was self-reported, recall bias may exist. Nevertheless, there were no official records, and we performed this study following the methods used in previous studies [24, 35]. Due to existence of such limitations, further studies are needed to clarify these issues.

## Conclusions

In the present cross-sectional study, we found that body composition was not significantly associated with LBP in both men and women, whereas height loss was a strong related factor of LBP in community-dwelling elderly individuals.

It was suggested that postural changes in elderly people due to height loss and the causative disease may lead to LBP whereas postural changes due to LBP may also cause height loss.

## Additional file

Additional file 1: **Table S1.** Correlation coefficients between different measures of body mass. *; *p* < 0.05, **; *p* < 0.01. (XLSX 25 kb)

Additional file 2: **Table S2.** A multiple linear regression analysis on height loss with the present body height and body weight. (XLSX 27 kb)

## Abbreviations

BIA: Bioelectrical impedance analysis
BMI: Body mass index
CI: Confidence interval
LBP: Low back pain
OR: Odds ratio
QUS: Quantitative ultrasound
SD: Standard deviation
YAM: Young adult mean
